# Splenomegaly – Diagnostic validity, work-up, and underlying causes

**DOI:** 10.1371/journal.pone.0186674

**Published:** 2017-11-14

**Authors:** Emelie Curovic Rotbain, Dennis Lund Hansen, Ove Schaffalitzky de Muckadell, Flemming Wibrand, Allan Meldgaard Lund, Henrik Frederiksen

**Affiliations:** 1 Department of Haematology, Odense University Hospital, Odense, Denmark; 2 Department of Medical Gastroenterology, Odense University Hospital, Odense, Denmark; 3 Department of Clinical Genetics, Copenhagen University Hospital, Rigshospitalet, Copenhagen, Denmark; European Institute of Oncology, ITALY

## Abstract

**Purpose:**

Our aim was to assess the validity of the ICD-10 code for splenomegaly in the Danish National Registry of Patients (DNRP), as well as to investigate which underlying diseases explained the observed splenomegaly.

**Background:**

Splenomegaly is a common finding in patients referred to an internal medical department and can be caused by a large spectrum of diseases, including haematological diseases and liver cirrhosis. However, some patients remain without a causal diagnosis, despite extensive medical work-up.

**Patients and methods:**

We identified 129 patients through the DNRP, that had been given the ICD-10 splenomegaly diagnosis code in 1994–2013 at Odense University Hospital, Denmark, excluding patients with prior splenomegaly, malignant haematological neoplasia or liver cirrhosis. Medical records were reviewed for validity of the splenomegaly diagnosis, diagnostic work-up, and the underlying disease was determined. The positive predictive value (PPV) with 95% confidence interval (CI) was calculated for the splenomegaly diagnosis code. Patients with idiopathic splenomegaly in on-going follow-up were also invited to be investigated for Gaucher disease.

**Results:**

The overall PPV was 92% (95% CI: 85, 96). Haematological diseases were the underlying causal diagnosis in 39%; hepatic diseases in 18%, infectious disease in 10% and other diseases in 8%. 25% of patients with splenomegaly remained without a causal diagnosis. Lymphoma was the most common haematological causal diagnosis and liver cirrhosis the most common hepatic causal diagnosis. None of the investigated patients with idiopathic splenomegaly had Gaucher disease.

**Conclusion:**

Our findings show that the splenomegaly diagnosis in the DNRP is valid and can be used in registry-based studies. However, because of suspected significant under-coding, it should be considered if supplementary data sources should be used in addition, in order to attain a more representative population. Haematological diseases were the most common cause, however in a large fraction of patients no causal diagnosis was found.

## Introduction

Splenomegaly is a common finding in patients referred to an internal medical departments and has been reported in 0.3% of all hospital admissions [1, 2] and amongst 2.9% of college freshmen [3] in North American populations. There is a large spectrum of underlying diseases and, as Osler wrote already in the beginning of the 20th century, splenic enlargement is almost always caused by diseases outside the spleen [4]. Consequently splenomegaly often requires extensive medical work-up from the diagnosing physician. Results from the few previous hospital-based cross-sectional studies that have been conducted in western countries 1996–1999 have showed large variation in the distribution of the underlying diagnosis [1, 2, 5]. Haematological diseases have been reported to account for 16–66%, hepatic diseases for 9–41%, infectious diseases for 9–36%, inflammatory or congestive diseases for 4–10%, primary splenic causes for 1–6%, and 1–2% remain idiopathic [1, 2, 5].

To the best of our knowledge, there is no published data on the risk of a subsequent diagnosis of haematological diseases, hepatic diseases or storage diseases, for patients already diagnosed with splenomegaly. Such data would help physicians in the diagnostic process of patients with splenomegaly, particularly in idiopathic cases when contemplating diagnostic splenectomy. Although splenectomy is a procedure that has become safer over time, risks remains [6], and recent studies have reported 20–52% incidence of postoperative complications and 1.2–2.4% postoperative mortality rates [7–9]. There is also an increased risk of thrombosis and infection following splenectomy [10]. The primary aim of this study was to investigate the data quality of the splenomegaly ICD10-diagnosis in the Danish National Registry of Patients (DNRP) by calculating the positive predictive value (PPV) for the diagnosis code. High registry validity would enable future registry-based research on the risks of being diagnosed with the disease groups stated above, following diagnosis of splenomegaly. The secondary aim was to describe the frequencies of causal diagnoses and the diagnostic work-up performed to make these. Furthermore we wanted to investigate if patients, that were classified by us as idiopathic splenomegaly, had Gaucher disease (GD).

## Materials and method

### Data source and study population

The patients included in this study were identified through the DNRP. The DNRP is a national health register and contains information of all non-psychiatric in-patient hospital admissions in Denmark since 1977 and all hospital out-patient specialist clinic visits since 1995 [11]. All Danish residents have been registered in the Danish Civil Registration System (CRS) since 1968 and given a 10-digit Civil Registration Number (CPR) [12]. All patients given an ICD10-diagnosis for splenomegaly (DR161, DR161A, DR162, DR162B and DQ890C) at Odense University Hospital (OUH) from January 1^st^ 1994 to December 31^st^ 2013 were identified through the DNRP. Patients with a prior ICD-8 diagnosis code of splenomegaly before January 1^st^ 1994 or patients with a known diagnosis of a malignant haematological neoplasia, or liver cirrhosis made before January 1^st^ 1994 were excluded. The CPR-numbers were used to link the patients to the medical records at OUH which provided clinical data.

A total of 129 patients met these criteria and their records were reviewed.

### Medical review record

All clinical records including laboratory result and medical imaging examinations were reviewed by ECR, and in selected cases HF and OSDM gave advice within their individual expert fields of haematology and medical gastroenterology, respectively. The splenomegaly diagnosis code was considered valid if one of the following criteria was met:

1. Clinically palpable spleen by abdominal examination on two occasions or by two physicians on the same occasion
2. The longest diameter being ≥13 cm by ultrasonography (US) [6] or >10 cm by computed tomography (CT) [13]
3. Wet weight >291 g at excision or autopsy [14]

Separate values were used for children according to age [15, 16]. Massive splenomegaly was further defined for all patients above the age of 15 years as one of the following:

1. Clinically palpable spleen >15 cm or >15 finger widths below the ribcage or at umbilicus level or lower
2. The longest diameter being > 18 cm on radiological imaging or described as greatly enlarged, or similar wording, by radiologist
3. Wet weight >1500 g at excision or autopsy

From the information in the clinical records the causal diagnoses were defined, if possible. These diagnoses were considered in reference to a relevant time span and an expert opinion was given in doubt of connection. Additional data were extracted from the records to characterise patients both clinically (presence of various symptoms, alcohol consumption etc.) and biochemically (total blood count, liver function etc.). Only biochemical results from one month prior or post to the splenomegaly diagnosis were included.

### Blood sampling

In a sub-study we investigated if patients, who after diagnostic work-up were classified by us as idiopathic splenomegaly, had GD. GD is a rare inherited lysosomal storage disease caused by a deficiency of the enzyme glucocerebrosidase. Clinical findings are highly variable and include splenomegaly; the diagnosis may be difficult as a result of rarity and variability of symptoms [17]. Patients that remained without a causal diagnosis, or with an uncertain causal diagnosis after review of medical record, were eligible for blood sampling.

Patients considered for blood sampling were evaluated individually and excluded if they had already (a) been biochemically tested for GD, (b) had a spleen that had regressed to normal size or (c) were discharged from follow-up at OUH. Based on these criteria, ten patients qualified for this procedure, and seven patients accepted the invitation to participate in blood sampling all of whom provided written informed consent.

Samples of EDTA-blood from all patients were analysed by AML and FW at Rigshospitalet, Copenhagen. GD diagnosis was based on activities of the enzymes glucocerebrosidase and chitotriosidase.

### Statistical analysis

The positive predictive value for the splenomegaly diagnosis was calculated with corresponding 95% confidence interval (CI). The Stata command diagt (STB-56: sbe36; STB-59: sbe36.1) was used to calculate PPV by dividing the number of patients with a valid diagnosis after review by the number of all patients. Descriptive tables were derived to illustrate the characteristics of the population in general and to compare differences across disease groups. P-values were calculated by using the chi-square test and the two-sample *t*-test. Data analyses were performed in STATA (StataCorp. 2015. *Stata Statistical Software*: *Release 14*.*1* College Station, TX: StataCorp LP).

### Ethics

Both studies were approved by the Danish Data Protection Agency (14/44365), and the Danish Health Authorities (3-3013-795/1/), and blood sampling was further approved by the Regional Committees on Health Research Ethics for Southern Denmark (S-20150063). All participants in the blood sampling substudy provided written informed consent.

## Results

Medical records were found for all 129 included patients. Mean age at the time of splenomegaly coding was 52 years and ranged from 0 to 91 years. 61% of the patients were male and 39% were female. The ICD-10 diagnosis code “Splenomegaly, not elsewhere classified” was used in 68% of the patients; “Splenomegaly, Not Otherwise Specified (NOS)” 22%, “Hepatomegaly with splenomegaly, not elsewhere classified” 9%, “Hepatosplenomegaly NOS” 1%. “Congenital splenomegaly” was never used.

### Validity of the splenomegaly diagnosis code

Out of 129 patients, 118 were correctly diagnosed with splenomegaly according to our criteria. Out of the 11 patients who were concluded to have been incorrectly diagnosed, six had normally sized spleens at ultrasound, according to our criteria, but had been considered enlarged at clinical examination. One was splenectomised due to idiopathic thrombocytopenic purpura and had a normal sized spleen at operation. Three were clearly coded with a splenomegaly diagnosis code entirely by mistake, and it was evident upon reviewing their records that a diagnosis code for a completely unrelated diagnosis, with a similar number but another letter, should have been used instead, such as E16.2 “Hypoglycaemia, unspecified” for example. One had undergone splenectomy for hereditary spherocytosis 60 years prior to splenomegaly coding. The PPV was calculated to 92% and increased during the study period from 87 (95% CI: 72–96) to 93 (95% CI: 86–98) (Table 1).

**Table 1:** Positive predictive values.

	Confirmed (total)	PPV (95% CI)
Overall	118 (129)	92 (85–96)
R161 “Splenomegaly, not elsewhere classified”	82 (88)	93 (86–98)
R161a “Splenomegaly NOS”	29 (29)	100 (88–100)
R162 “Hepatosplenomagly NOS”	1 (1)	100 (2–100)
1994–2003	33 (38)	87 (72–96)
2004–2013	85 (91)	93 (86–98)
0–50 years	53 (55)	96 (88–100)
51–100 years	65 (74)	88 (78–94)
Males	74 (79)	94 (86–100)
Females	44 (50)	88 (76–96)

Positive predictive values for ICD-10 diagnosis codes in the DNPR. The lowercase suffix on 161a is a specific part of the Danish implementation of ICD-10.

The splenomegaly diagnosis was confirmed for the first time by US in 60% of the patients, CT in 27%, palpation in 10%, other method of examination 2%, and excision in 1%. One case was confirmed during explorative laparotomy, performed due to ileus. The median time elapsed between the date when splenomegaly was confirmed at our review and the date of the coding for splenomegaly was seven days (range: -6 to 248 months).

### Underlying causal diagnoses

The mean time from the date of splenomegaly diagnosis to the date of the underlying diagnosis was five months and ranged from -10 to 19 years. Table 2 describes the distribution of causal diagnoses for all patients with splenomegaly and patients with massive splenomegaly. Within the ICD-10 splenomegaly group, haematological diseases were the most common causes, followed by hepatic diseases (Table 2). A fourth of the patients had no diagnosis that explained their splenomegaly. Among patients with massive splenomegaly, haematological diseases were the most common causal diagnosis (64%), while 13% had no underlying diagnosis.

**Table 2:** Causal diagnoses.

	All splenomegaly		Massive splenomegaly^a^	
	n = 118		n = 46	
	N	%	n	%
**Diagnostic group**				
***Haematological***	47	39	29	64
Lymphoma	20	17		
MPN	16	14		
CLL/HCL	6	5		
Haemolytic diseases	2	2		
Other haematological diseases	3	3		
***Hepatic***	21	18	6	13
Liver cirrhosis	13	11		
Portal vein thrombosis	4	3		
Portal hypertension, other cause than thrombosis	3	3		
Cancer	1	1		
***Infectious***	12	10	1	2
Acute mononucleosis^a^	4	3		
CMV^b^	2	2		
Endocarditis	1	1		
Unidentified infection	6	5		
***Primary splenic***	3	3	2	4
***Inflammatory***	2	2	1	2
***Other diseases***	3	3	1	2
***Idiopathic / unknown***	30	25	6	13

Causal diagnoses, divided in diagnostic groups, for splenomegaly and massive splenomegaly.

^a^ Only defined for patients ≥15 years old

^b^ One patient presented with both acute mononucleosis and CMV

**Fig 1:**
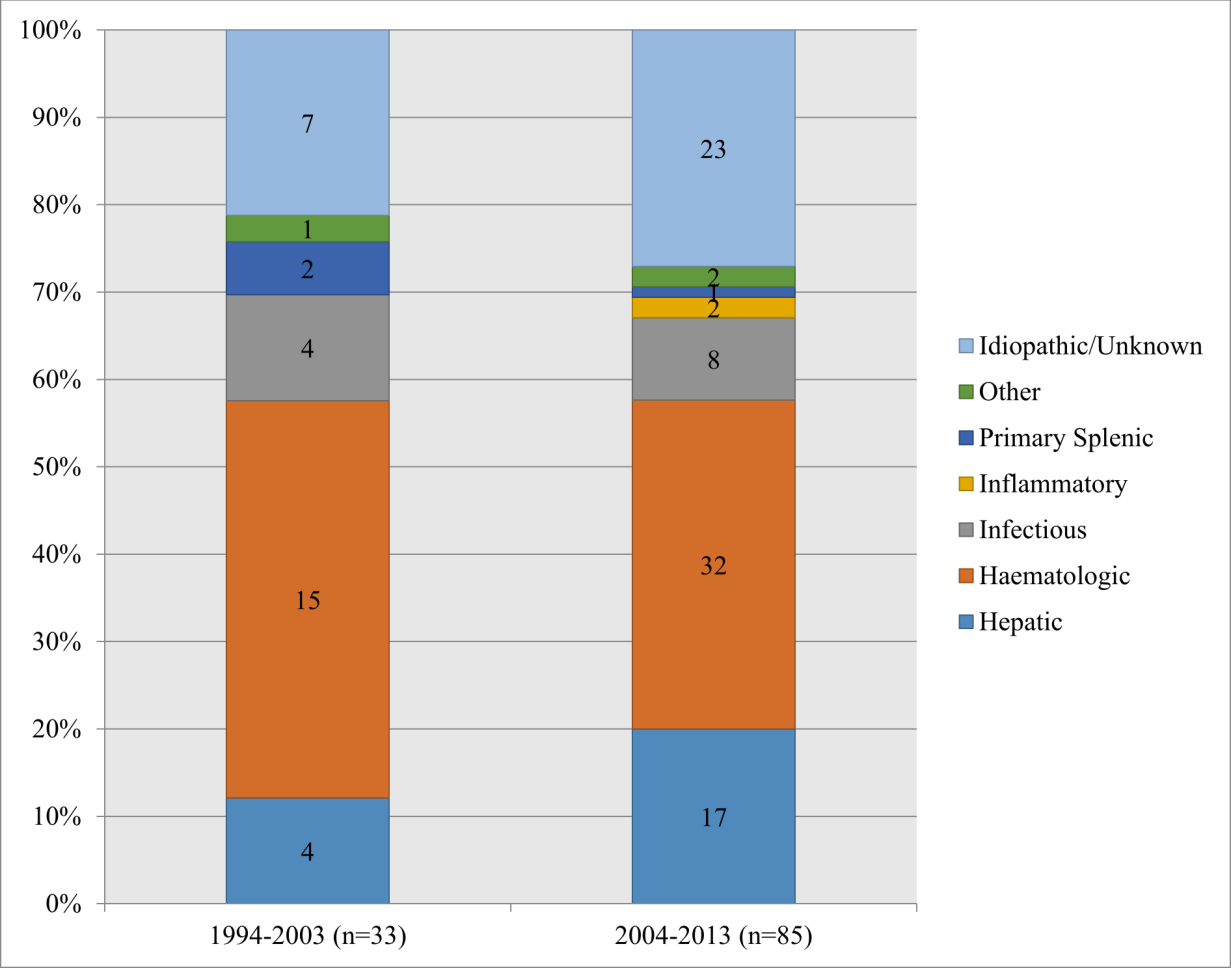
Distribution of diagnostic groups. Distribution of diagnostic groups for patients diagnosed with splenomegaly over the time periods 1994–2003 and 2004–2013.

The distribution of diagnostic groups, comparing patients diagnosed with splenomegaly in the years of 1994–2003 (1^st^ decade) and 2004–2013 (2^nd^ decade), can be seen in Fig 1. The total number of patients diagnosed in the 2^nd^ decade (85) is almost three times as large as in the 1^st^ decade (33). The total amount of patients increased over time in all diagnostic groups, except the primary splenic group.

Among the haematological diseases lymphomas accounted for 43%, myeloproliferative neoplasms (MPN) 34%, and chronic lymphocytic leukaemia (CLL)/hairy cell leukaemia (HCL) 13% (Table 2). Liver cirrhosis was the cause of 62% of the hepatic diagnoses and all these patients were diagnosed with splenomegaly during the 2^nd^ decade.

Only 6% of the haematological patients were coded with splenomegaly for the first time at the Department of Haematology; the largest part (53%) was coded at the Department of Surgery. Nineteen percent of the hepatic group were coded with splenomegaly for the first time at the Department of Gastrointestinal Diseases.

### Features of diagnostic groups

A comparison of clinical signs, laboratory values and characteristics between the hepatic, haematological and infectious group can be seen in S1 Table. About half of the patients with hepatic diagnoses had hepatomegaly, clinical signs of liver disease and thrombocytopenia and all were significantly more common than in the haematological group and the infectious group. The haematological group had the highest percentage of massive splenomegaly. Likewise, the haematological group had the largest mean spleen length (S1 Table). A total of 42% of the patients had massive splenomegaly (95% CI 32–51). The infectious group were more likely than others to have fever (p<0.05) and lymphadenopathy.

### Diagnostic work-up

**Fig 2:**
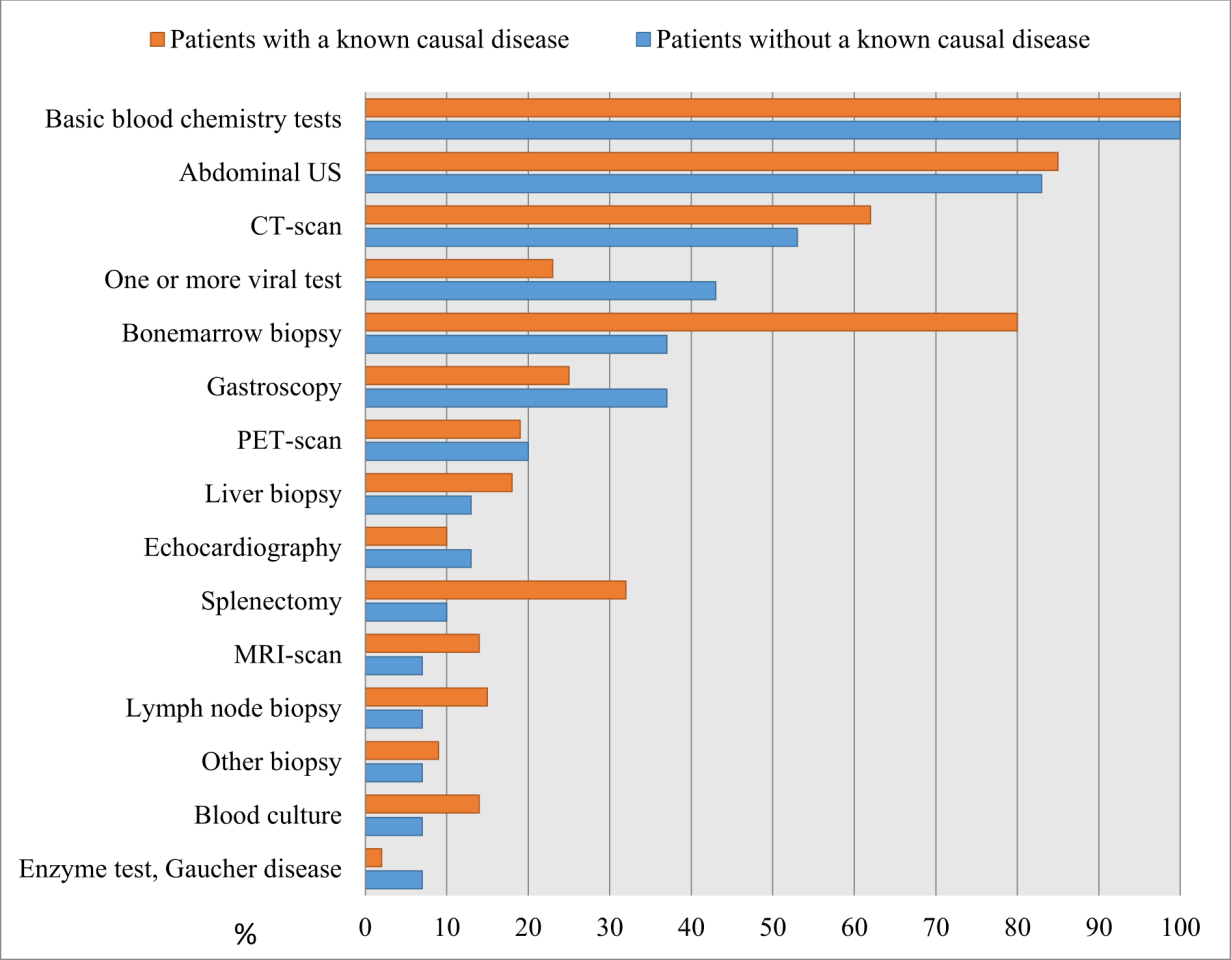
Diagnostic work-up. The percentages of patients that underwent different diagnostic procedures during the investigation.

Fig 2 displays the diagnostic procedures performed during diagnostic work-up. All patients had basic blood chemistry tests performed and 83% of the idiopathic patients and 85% of the diagnosed patients were examined with an US. One or more test for viral disease was performed on 43% of the idiopathic patients and 23% of the diagnosed patients. Just over a third (37%) of the idiopathic patients had a bone marrow biopsy taken, compared to 80% of the diagnosed patients. Ten percent of the idiopathic patients and 32% of the diagnosed patients were splenectomised, where the indication for surgery could be diagnostic as well as therapeutic. Lymph node biopsies were about twice as common among the diagnosed patients (15%) as among the idiopathic patients (7%). Seven percent of the idiopathic patients and 2% of the diagnosed patients were tested for GD before inclusion in our study. Of the ten patients with idiopathic splenomegaly who were eligible for blood sampling seven participated, all of whom tested negative for GD.

### Splenectomy

Table 3 illustrates that a total of 26% were splenectomised for all causes, both diagnostic and therapeutic. The largest proportion of splenectomised patients was found in the haematological group (47%). The incidence of splenectomy in the haematological group increased from 20% of the patients diagnosed in the 1^st^ decade to 59% in the 2^nd^ and in the hepatic it decreased from 25% to 6%. More than half of the removed spleens were massively enlarged.

**Table 3:** Splenectomised.

	All patients	Hepatic	Haematological	Infectious	Other
	%	%	%	%	%
Splenectomised, total	26	10	47	0	18
Splenectomised, diagnosed in 1^st^ decade	18	25	20	0	20
Splenectomised, diagnosed in 2^nd^ decade	29	6	59	0	18
Splenectomised + massive splenomegaly^a^	15	0	29	0	11

Percent splenectomised in all and per group out of the 118 correctly diagnosed patients. Other includes the primary splenic group, inflammatory group, various group and idiopathic group. Decades refers to if the patient was diagnosed with splenomegaly in the 1^**st**^ or 2^**nd**^ decade of the study period.

^**a**^Patients ≥15 years old)

## Discussion

Our study demonstrates that the ICD-10 splenomegaly diagnosis in the DNRP is valid and that the registry diagnosis can be used without further validation in future research. Many studies have been conducted to evaluate the validity of different ICD-10 diagnoses in the DNRP. Some of the most recent studies that have also used medical records as a gold standard have reached varying results for the PPV; haemolysis 87.1% [18], gram-negative septicaemia/sepsis or urosepsis 72% [19], cardiogenic, hypovolemic, and septic shock 86.1% [20] and undernutrition 70.9% [21].

The delay between the finding of splenomegaly and coding is relatively short. It is likely, however, that when a causal diagnosis is evident a splenomegaly finding is not always associated with a splenomegaly ICD diagnosis code. Therefore the splenomegaly finding is probably under-coded in the ICD system and the total amount of patients coded during our study period was quite small. The distribution of departments first coding patients with a splenomegaly ICD-10 code emphasizes this since only a small part of the patients with a haematological or hepatic causal disease were coded with a splenomegaly code at their corresponding specialised departments. Thirty-four percent of all patients and 53% of haematological patients were coded at the Department of Surgery; most were admitted there either due to abdominal pain or for planned splenectomy. Also, patients from all peripheral hospitals in the Region of Southern Denmark are referred to OUH for splenectomy, and it is therefore possible that they had been coded with the causal diagnosis at their regional hospitals prior to referral to the Department of Surgery at OUH.

All of this suggests that, in order to include a more complete group in a study of patients with splenomegaly, other modalities, such as radiological records, are required. Radiologists in Denmark do not associate their findings with codes in the DNRP, but identifying patients with splenomegaly through their text records may be feasible.

### Causal diseases and unexplained splenomegaly

The distribution of causal diseases for both all splenomegaly cases and massive splenomegaly cases is in concordance with that of previously published studies, except for the high occurrence of idiopathic splenomegaly [1, 2, 5]. Unexplained massive splenomegaly, was not reported in previous studies, but was seen in 13% of patients with massive splenomegaly in our study. It must however be considered that our population, due to suspected undercoding, might not be representative for patients with splenomegaly in general. The overall proportion of patients with massive splenomegaly in our study was much higher (42%) than in the prior modern western studies (21–27%) [1, 2, 5], indicating that our splenomegaly population could be more severely ill than the populations in preceding studies. A previous study of patients with non-alcoholic fatty liver has shown a positive correlation between the degree of fatty infiltration in the liver and the spleen size [22], demonstrating that spleen size may correlate to the severity of illness.

### Features of diagnostic groups and diagnostic evaluation

Clinical and para-clinical features across causal diagnostic groups were largely as expected. However, enlarged lymph nodes were more common in the infectious group than in the haematological group, which has not been seen in previous studies [1, 2, 5]. An algorithm derived by Eichner and Whitfield suggests that lymphadenopathy should lead to examination for CLL, lymphomas and granulomatous diseases [23]. Though the percentage of patients with lymphadenopathy was higher in the infectious group than in the haematological group, the total amount of patients who had splenomegaly, lymphadenopathy and a haematological disease was three times the number of patients who had splenomegaly, lymphadenopathy and an infection. This indicates that haematological diseases should be excluded for patients, especially the elderly, with lymphadenopathy, before settling with a diagnosis of infection. The finding of massive splenomegaly should likewise lead to a thorough haematological investigation.

The displayed difference in characteristics, presence of clinical signs and laboratory results between diagnostic groups show that a thorough clinical history, physical examination and blood tests remain important in order to plan the diagnostic work-up. In our study patients classified with idiopathic splenomegaly generally had less, and possibly insufficient, diagnostic work-up. For ethical reasons, only patients in on-going follow-up were eligible for blood sampling for GD. All patients tested negative, ruling out GD among a third of the patients with idiopathic splenomegaly. Due to this small sample it is however, difficult to draw any conclusions from these results, and GD should still be considered as a differential diagnosis.

## Conclusion

The ICD-10 splenomegaly diagnosis code is valid and can be used in future registry-based research. However, because of suspected significant under-coding, it should be considered if text data from radiological reports can be used to maximise the number of patients with splenomegaly included in a future study population. We have also shown that many patients remain without an explanatory diagnosis. Certain clinical and laboratory data were associated with different disease groups and may be used to guide physicians in their diagnostic investigations.

## Supporting information

S1 Table Clinical signs, characteristics and laboratory values. Clinical signs, characteristics and laboratory values associated with diagnostic groups. HPG = Hepatic group (n = 21), HMG = Haematological group (n = 47), IG = infectious group (n = 12) and All = all patients with splenomegaly (n = 118). The number of patients where data was found is specified for each symptom/laboratory value/characteristic. Laboratory tests included samples taken between 30 days prior to and 30 days after splenomegaly coding, from blood (B) or plasma (P).
(DOCX)
