# Supplementary material for: Splenomegaly – Diagnostic validity, work-up, and underlying causes
Source: PLoS One. 2017 Nov 14;12(11):e0186674. doi: 10.1371/journal.pone.0186674 (PMC5685614; doi:10.1371/journal.pone.0186674)
Supplement: S1 Table — Clinical signs, characteristics and laboratory values associated with diagnostic groups. HPG = Hepatic group (n = 21), HMG = Haematological group (n = 47), IG = infectious group (n = 12) and All = all patients with splenomegaly (n = 118). The number of patients where data was found is specified for each symptom/laboratory value/characteristic. Laboratory tests included samples taken between 30 days prior to and 30 days after splenomegaly coding, from blood (B) or plasma (P). (DOCX) [file pone.0186674.s001.docx]

| **Diagnostic Group** | **HPG (n=21)** | | **HMG (n=47)** | | **IG (n=12)** | | **All (n=118)** | |
| --- | --- | --- | --- | --- | --- | --- | --- | --- |
|  | n | % | n | % | n | % | n | % |
| Hepatomegaly (n=118) | 11 | 52 | 9 | 19 | 2 | 17 | 30 | 25 |
| Lymphadenopathy (n=117) | 5 | 24 | 18 | 38 | 6 | 55 | 34 | 29 |
| Pain/tenderness/filling in the upper left quadrant (n=116) | 7 | 33 | 21 | 46 | 2 | 18 | 42 | 36 |
| Fever (T>38 degrees C ≥2 days) (n=117) | 1 | 5 | 8 | 17 | 7 | 58 | 23 | 20 |
| Night sweating (n=118) | 0 | 0 | 8 | 17 | 3 | 25 | 16 | 14 |
| Weight loss (≥ 10% in 6 months) (n=118) | 5 | 24 | 11 | 23 | 4 | 33 | 26 | 22 |
| Clinical signs of liver failure (n=118) | 11 | 52 | 5 | 11 | 2 | 17 | 23 | 19 |
| Alcohol overconsumption (n=102) | 3 | 18 | 3 | 7 | 0 | 0 | 7 | 7 |
| Sex, male (n=118) | 13 | 62 | 25 | 53 | 11 | 92 | 74 | 63 |
| Haemoglobin < 11.76/13.37 g/dL for F/M (n=105)^a^ | 11 | 61 | 26 | 59 | 5 | 56 | 61 | 58 |
| Thrombocytes < 100*10^9^ / L (n=112) | 10 | 50 | 11 | 24 | 0 | 0 | 27 | 24 |
| Leucocytes < 3.5*10^9^ / L (n=96)^b^ | 1 | 6 | 4 | 10 | 0 | 0 | 9 | 9 |
| Leucocytes > 8.8*10^9^ / L (n=96)^b^ | 1 | 6 | 21 | 51 | 3 | 38 | 33 | 34 |
| Albumin <36/34 g/L for age 15-70/70 and older (n=100)^a^ | 8 | 44 | 7 | 17 | 2 | 22 | 27 | 27 |
| LDH >205/255 U/L for age 18-70/70 and older (n=91)^b^ | 9 | 53 | 24 | 67 | 5 | 62 | 53 | 58 |
| Bilirubin >25 µmol/L (n=102) | 4 | 20 | 12 | 32 | 2 | 18 | 21 | 21 |
| Massive splenomegaly (n=100) | 6 | 32 | 29 | 64 | 1 | 10 | 46 | 42 |
|  | Mean | 95%CI | Mean | 95%CI | Mean | 95%CI | Mean | 95%CI |
| Spleen length at first evaluation of length, cm (n=96)^a^ | 17.2 | 15.7-18.6 | 20.1 | 18.3-21.9 | 15.7 | 14.4-17.0 | 18.1 | 17.2-19.0 |
| Age at 1^st^ splenomegaly coding, years (n=118) | 46 | 38-55 | 64 | 59-70 | 29 | 16-42 | 51 | 47-55 |
| Haemoglobin, g/dL (n=105) | 12.09 | 11.12-12.88 | 11.84 | 11.27-12.72 | 13.2 | 11.91-14.33 | 12.24 | 11.75-12.56 |
| Thrombocytes, *10^9^ / L (n=112) | 124 | 83-166 | 184 | 130-239 | 211 | 171-251 | 184 | 155-213 |

^a^Patients <15 years excluded, total patients (n=110)

^b^Patients <18 years excluded, total patients (n=104)
